# Separation of CH_4_/N_2_ of Low Concentrations From Coal Bed Gas by Sodium-Modified Clinoptilolite

**DOI:** 10.3389/fchem.2018.00633

**Published:** 2018-12-18

**Authors:** Xiaofei Hao, Zhen Li, Hongjie Hu, Xueqin Liu, Yanqiu Huang

**Affiliations:** ^1^Faculty of Materials Science and Chemistry, China University of Geosciences, Wuhan, China; ^2^Zhengzhou Fulong Science and Technology of New Materials Co., Ltd., Zhengzhou Institute of Multipurpose Utilization of Mineral Resources, Chinese Academy of Geological Sciences, Zhengzhou, China

**Keywords:** clinoptilolite, ion-exchange, nitrogen/methane separation, selectivity, low concentrations methane, pressure swing adsorption

## Abstract

Clinoptilolite is a widely distributed tectosilicate, mainly composed of Al_2_O_3_, SiO_2_ with exchangeable cations such as Ca, K, Mg, and Na. In this research, raw clinoptilolite was ground, gravimetrically concentrated and ion-exchanged using different concentrations of NaCl solution. Then the modified clinoptilolite powder was formulated into particles as adsorbents. The adsorbents were applied to CH_4_ separation in coal bed gas. The raw and modified clinoptilolites were characterized by X-ray diffraction (XRD), scanning electron microscope (SEM), transmission electron microscope (TEM), atomic emission spectrometer (ICP-AES), Fourier transform infrared spectrometer (FTIR), and Brunauer Emmett Teller (BET) specific surface area. The CH_4_ absorptivity by raw and modified clinoptilolites was evaluated using pressure swing adsorption (PSA) to assess the CH_4_ separation ability. The results indicated that the ion-exchanged clinoptilolite using 0.2 mol/L NaCl solution was found to be promising for the kinetic PSA separation of CH_4_/N_2_, giving a better absorptivity for CH_4_ separation under different influence factors. Based on the simulated static experiments, it was indicated that both CH_4_ and N_2_ were capable of diffusing into clinoptilolite while N_2_ adsorption by clinoptilolite was excellent. The experiment results also indicated that ion-exchanged clinoptilolite using a 0.2 mol/L NaCl solution was the optimal adsorbent for separating CH_4_/N_2_ at the low pressure condition. From the simulated dynamic experiments, the ion-exchanged clinoptilolite using a 0.2 mol/L NaCl solution as a potential sorbent in kinetic PSA processes for N_2_/CH_4_ separation, exhibited the best performance at 648 K under 0.2 MPa within 28 min, in comparison to the raw clinoptilolite and clinoptilolite under other modification conditions. In the next phase of research, the modified clinoptilolite will be tested for CH_4_ separation in real coal bed gas.

## Introduction

The coalbed gas is found in coal bed with a main composition of methane (CH_4_), which was absorbed on the surface of the coal particles. Part of coal bed gas was dissociated or dissolved in the hydrocarbon gas in the coal pore and the water of coal bed, which is automatically stored up in the coal bed as the powerful complement to raw gas. CH_4_ in the coal bed is a high quality gas fuel. Meanwhile, it is also one of detrimental gases influencing mining underground coal and an important harmful source leading to atmospheric greenhouse effect. In China, there is up to 13 billion m^3^ CH_4_ under the process of coal mine a year, which accounts for around one third of its emissions globally. On the other hand, the utilization ratio of CH_4_ in the coal bed gas was only 35%, resulting in a huge CH_4_ resource loss. We know that the greenhouse effect of CH_4_ is 21 times to the CO_2_ and power of CH_4_ for damaging ozone (O_3_) is 7 times to the CO_2_. Thus, recycling coal bed gas is of great significance on both energy development and environmental protection. With improving consciousness of human on the coal mine safety and environmental protection, the exploitation of CH_4_ in the coal bed has been attached great importance to the world in recent years.

The separation technology of CH_4_ in the coal bed is not effective, which is one of the main reasons for low the utilization ratio. In the separation process of low concentration of coal bed gas, the physicochemical property of N_2_ and CH_4_ was similar (Perry et al., 1999; Johnson III, 2015). It led the recycle and separation technology to be a key common technology challenge. It was also one of the most important technological obstacles on gas development, energy saving and emission reduction (Bomberger et al., 1999; Cavenati et al., 2006; Tagliabue et al., 2009). At present, the common technologies were cryogenic distillation, pressure swing adsorption (PSA), membrane separation, hydration technology and dissolution-absorption technology. The PSA separation method has become the mainstream technology for the purification of coal bed gas at small and medium scales, due to its advantages of low energy consumption, less investment equipment and high degree of automation (Arya et al., 2014; Yin et al., 2015). Its key challenge is the selection of adsorbents. The main adsorbents currently used are activated carbon (AC) (Zhou et al., 2002; Gu et al., 2015; Gao et al., 2017), carbon molecular sieve (CMS) (Fatehi et al., 1995; Cavenati et al., 2005; Grande et al., 2005), natural clinoptilolite (Aguilar-Armenta et al., 2001; Jayaraman et al., 2004, 2005), titanium silicon molecular sieve (Aguilar-Armenta et al., 2001; Jayaraman et al., 2004, 2005; Faghihian et al., 2008). The equilibrium adsorption capacity of CH_4_ is higher than that of N_2_ for AC. Although the separation coefficient is higher and the effect is better based on results from laboratory studies, it is still far away from industrial application. The main reason is that the preparation process of AC is complex and the cost is relatively high. And it obtains CH_4_ product in vacuum desorption stage, the subsequent operation needs to be compressed, so the power cost is increased, and the economic effect is not obvious. The separation of CH_4_ and N_2_ by CMS is based on the kinetic effect. The diffusion rate of N_2_ in the micropore is higher than CH_4_. A large amount of N_2_ is adsorbed into the pore and CH_4_ remains outside the pore in a relatively short time. Therefore, the product CH_4_ is obtained by the adsorption or sequestration of the PSA process, instead of the vacuum step. With the increase of adsorption time, the kinetically the effect becomes weaker, and the equilibrium effect will dominate, making CH_4_ and N_2_ separation difficult. Although CMS has achieved good results in the laboratory, it is mainly aimed at the coal bed gas with high concentration of CH_4_ (CH_4_ content >70%). However, there are few reports on the study of coal bed gas with low concentrations. The natural clinoptilolite as a kind of PSA adsorbents is of great potential for application with the advantage of acid resistance, heat resistance, alkali resistance, stable structure, rich resources, and low price. They can show both equilibrium and kinetic effects. However, the adsorbent prepared from natural clinoptilolite are of different sodium contents and its application in low concentration coal bed gas (CH_4_ < 30%) has not been reported.

Here we present a study of the adsorption isotherms of four adsorbents made from natural clinoptilolite with different sodium contents at 298 K. And the corresponding adsorption kinetics were measured at the same and different pressure using the feed gas containing 20% CH_4_ and 80% N_2_ at 298 K. This study will provide technical support for the implementation of industrialization.

## Materials and Methods

### Materials

#### Clinoptilolite and All Agents

The raw clinoptilolite was collected from the south of the Liaoxi metallogenic belt in China. The adsorbents used were CH_4_ (99.95%), N_2_ (99.95%). The purging gas for adsorbent activation/regeneration was He (99.999%, pre-purified). All gases were provided by Praxair. The reagents, including NaCl, used in this research were of analytically pure and bought from Sinopharm Group Chemical Reagent Co., Ltd.

#### Clinoptilolite Pretreatment

The raw clinoptilolite was ground by a ball grinder to a granular size <70 μm. Then the milled pulp with clinoptilolite powders was poured into a Falcon centrifuge to remove some heavy impurities. The purified clinoptilolite was dried at 105°C and stored in a desiccator. It was used as a raw material for the preparation of adsorbents.

#### Preparation of Modified Clinoptilolite

The processed clinoptilolite powders were mixed NaCl solutions at concentrations of 0.1, 0.2, 0.4, and 0.6 mol/L at a solid to liquid ratio of 1:20 for 2.5 h in Erlenmeyer flasks separately and covered with sealing films and maintained in a 90°C water bath. The mixture was centrifuged to separate the solids, then washed using deionized water until no Cl^−^. All ion-exchanged clinoptilolite samples were pressed into a round cake and calcined at 200°C (to dry the samples) for 2 h. And then they were crushed and sieved. Particles of 0.5–1.5 mm sizes were used as adsorbents.

#### Separation Experiment With CH_4_/N_2_

**Figure 1:**
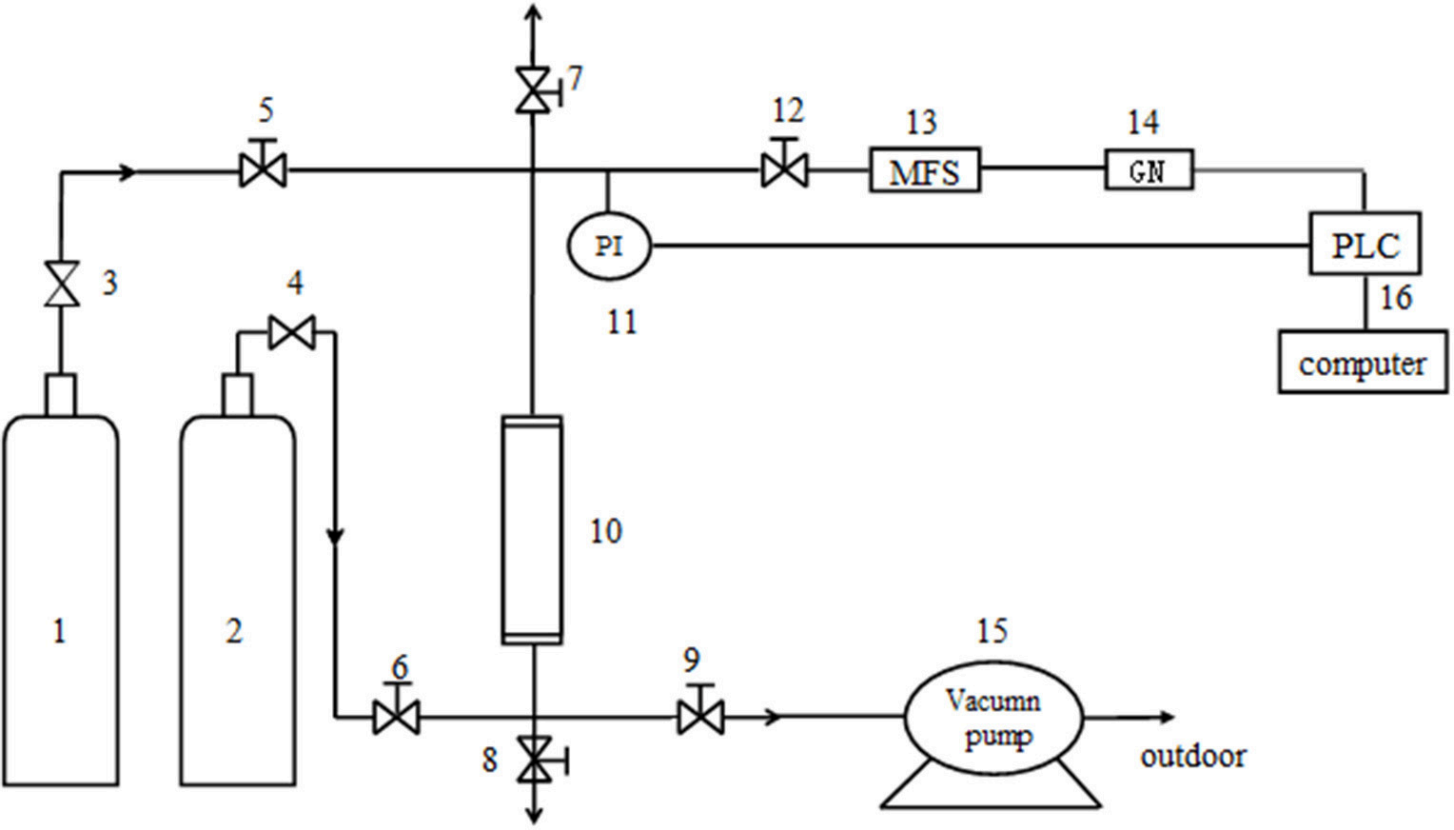
The dynamic test device. 1. He cylinder; 2. Mixture cylinder of CH_4_/N_2_; 3, 4. Pressure reducing valve; 5, 6, 7, 8, 9 and 12. Needle-type valve; 10. Absorbing tower(16mm^*^320mm); 11. Pressure probe; 13. Mass flow controller; 14. Gas analyzer; 15. Vacuum pump; 16. Windows control center (Copyright^©^1994–1997 SIEMENS, AG).

The gas mixture of CH_4_ and N_2_ was prepared by high-pure standard gas, and the ratio of CH_4_/N_2_ was 20/80%. The experimental device for adsorption was a single absorbing tower filled with raw and modified clinoptilolites (Figure 1). At first, the device was pressurized using high-pure standard He until the adsorption pressure was up to the setting pressure. Then the intake valve of He was closed and the intake valve of mixture gas was opened (it was the start time of data recording). In order to keep the pressure of absorbing tower reaching the experimental value, it was adjusted by control valves (the flow value of gas was set to 50 mL/min). The outlet discharge was set using mass flow controller before the test. In the process of absorption, the change of concentration of CH_4_ was tested and recorded by a gas analyzer. The test was continued until the concentration of CH_4_ was the same as the initial concentration of CH_4_ in the mixture. The activation and regeneration of modified clinoptilolite was not begun until inverse vacuum was pumped for 10 min.

#### Pressure/Vacuum Swing Adsorption Experimental Program

A vacuum pressure swing adsorption (VPSA) cycle was devised to be experimented in the pilot-scale unit, which was the purpose of catching CH_4_ from a dynamical mix of CH_4_/N_2_ simulated coal bed gas. The device, single-tower adsorption layer, was shown in Figure 1. Before test, the absorbents were modified clinoptilolites which were activated in the vacuum rotation activation furnace for 6 h in 648 K. When the test began, the pressure of He and pressure of adsorption were both set at the certain pressure, and flow value of tower top was 60 mL/min. It needed to be emphasized that vacuum pumping treatment using vacuum pumps was initiated before the experiments.

### Analytical Methods

The morphologies of purified clinoptilolite and modified clinoptilolite were observed with TEM (Tecnai G2 TF30) and SEM (Hitachi S-4800).

X-ray diffraction (Rigaku MiniFlex600) measurements were proceeded with copper CuKα1 radiation (λ = 1.5406 Å), utilizing a voltage of 40 kV and a current of 15 mA. The divergence slit was 0.3 mm and data was gathered for 2θ scanned from 3° to 80° at 10°/min.

The chemical components of purified clinoptilolite and modified clinoptilolite samples were analyzed using an inductively coupled plasma atomic emission spectroscopy (ICP-AES, ICAP7400 THERMO Fisher). To obtain a more representative chemical composition of a sample, the analysis was done in triplicates per sample, and the element contents were averaged (Table 1).

**Table 1:** The chemical composition of clinoptilolites (wt%).

**Composition (wt %)**	**Modified clinoptilolites**				
	**C-0**	**C-1**	**C-2**	**C-3**	**C-4**
SiO_2_	66.99	68.49	68.48	68.08	68.09
Al_2_O_3_	12.01	12.86	12.85	13.02	13.01
K_2_O	1.63	1.65	1.62	1.61	1.59
Na_2_O	0.65	1.94	2.45	3.30	3.68
CaO	3.80	3.09	2.73	2.14	1.89
MgO	1.29	1.26	1.20	1.08	1.01
Fe_2_O_3_	1.37	1.47	1.48	1.50	1.60
TiO_2_	0.20	0.19	0.19	0.19	0.19

Mid-infrared spectra were recorded using a Fourier transform infrared (FT-IR) spectrometer (Nicolet IS50) with a Smart Endurance™ single bounce diamond ATR cell. Spectra were acquired of 4,000–400 cm^−1^ by the average of 64 scans with a resolution ratio of 4 cm^−1^. A mirror speed of 0.6 cm/s was used.

The BET surface area was 57.84 ± 0.20 m^2^/g measured by an ASAP 2020 instrument (Micromeritics, USA). N_2_ (at 298 K) and CH_4_ (at 298 K) adsorption were measured to determine the BET surface area and micropore size distribution.

## Results and Discussion

### Characterization of Clinoptilolites

**Figure 2:**
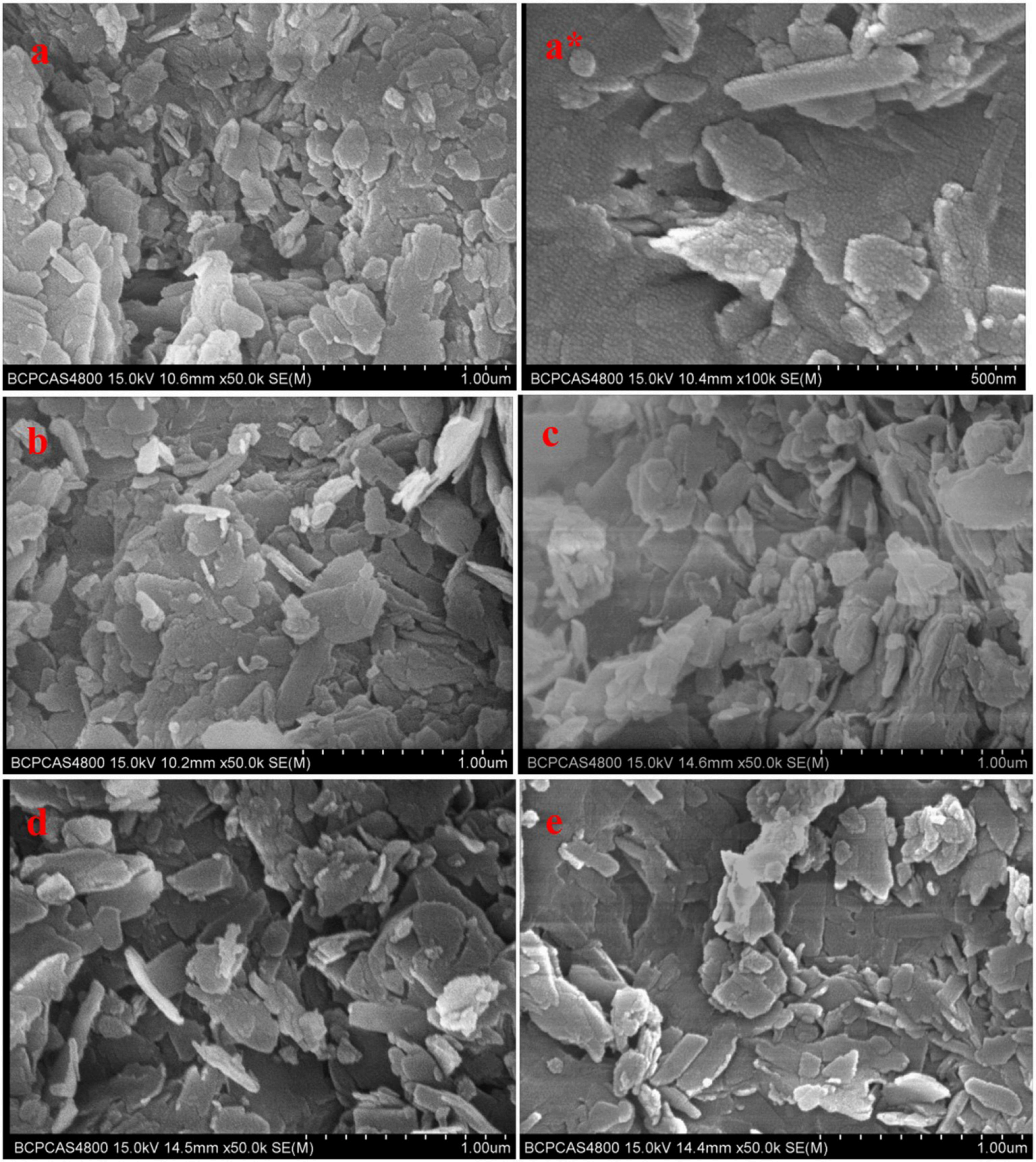
SEM photographs of clinoptilolites: **(a**,a*). C-0, **(b)**. C-1, **(c)**. C-2, **(d)**. C-3, **(e)**. C-4.

Figure 2 showed SEM photographs of unmodified and modified clinoptilolites, They didn't change significantly among them. In Figure 2, C-0, unmodified clinoptilolite, was purified clinoptilolite with no NaCl treatment. The modified clinoptilolites were depicted by C-1, C-2, C-3, and C-4, which were treated by 0.1, 0.2, 0.4, and 0.6 mol/L NaCl solution, respectively. These crystals are flaggy or schistose, which appears as parallel conjunctive aggregate. Individual crystals span is from hundreds of nanometers to several microns. A number of ordered small particles were also found on the crystals from Figure 2a^*^.

**Figure 3:**
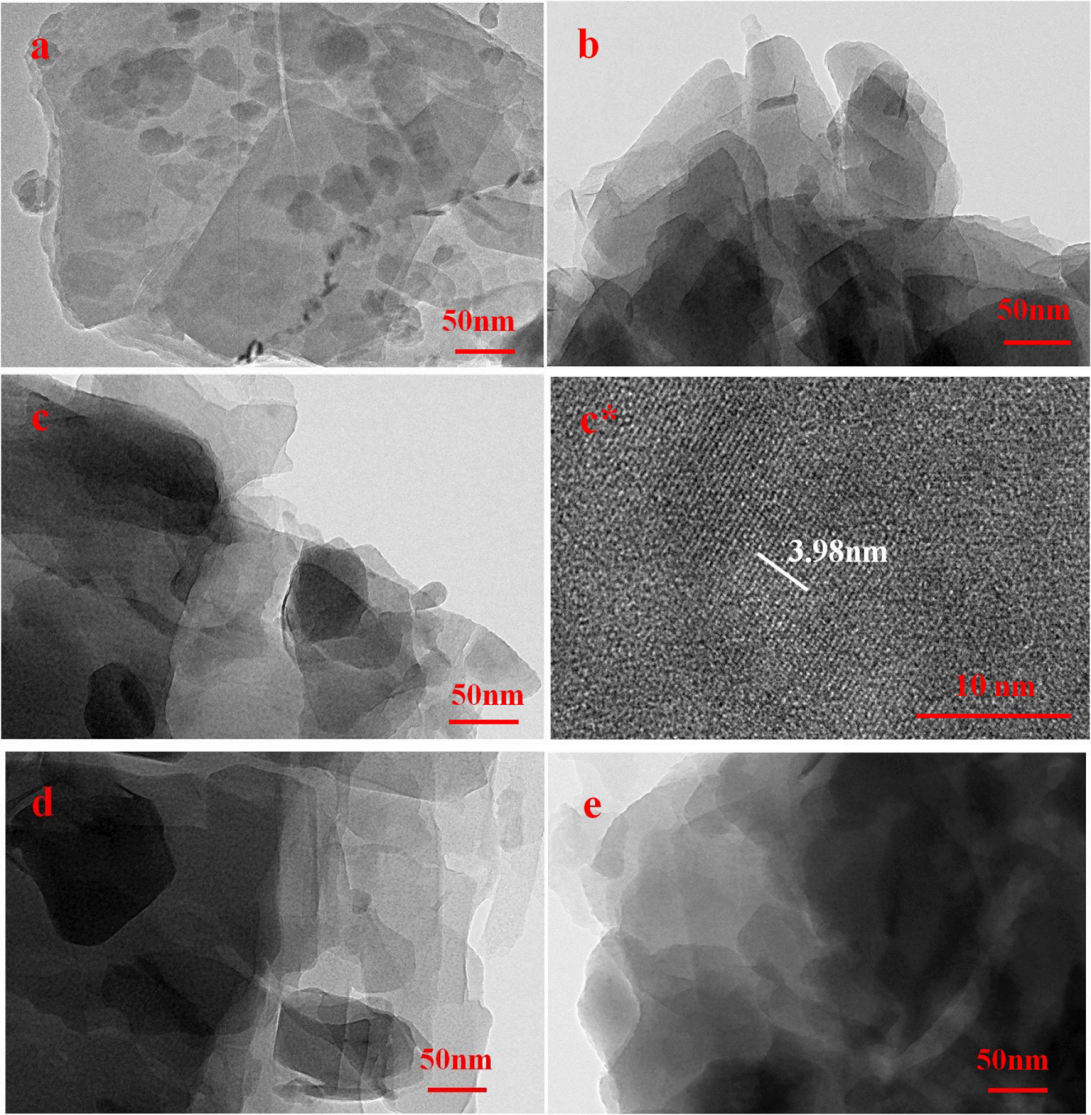
TEM photographs of clinoptilolites: **(a)**. C-0, **(b)**. C-1, (c,c*). C-2, **(d)**. C-3, **(e)**. C-4.

In order to observe the internal microstructure of samples, TEM photographs of modified clinoptilolites were shown in Figure 3. From Figure 3, inside of C-0 was large lamella stacking. However, inside of C-1, C-2, C-3, and C-4 were stacked with small lamella, in which the number of fissures produced by flake particles. This is because they are stirred on thin sheets during ion exchange, so more lamellar clinoptilolite is stripped, mainly concentrated at 100 × 300 nm, which also makes the exchange easier. The 131 faces of clinoptilolite can be seen in C-2 of photograph c^*^, whose crystal plane spacing is 3.98 Å, which have not seen in other published papers.

ICP-AES was adopted to analyze the ion change before and after modification (table 1). It is obviously that with the increasing of nacl concentrations, the Na^+^ component increased from 0.65 to 3.68 wt% gradually. Meanwhile, the Ca^2+^ component decreased from 3.8 to 1.89 wt% apparently. In addition, other elements showed a random change with increased nacl concentrations (such as Si, Al, K, Mg, Fe, and Ti). The phenomenon indicated that the ion exchange of Na^+^ for Ca^2+^ were carried out in clinoptilolite samples with NaCl solutions bath.

**Figure 4:**
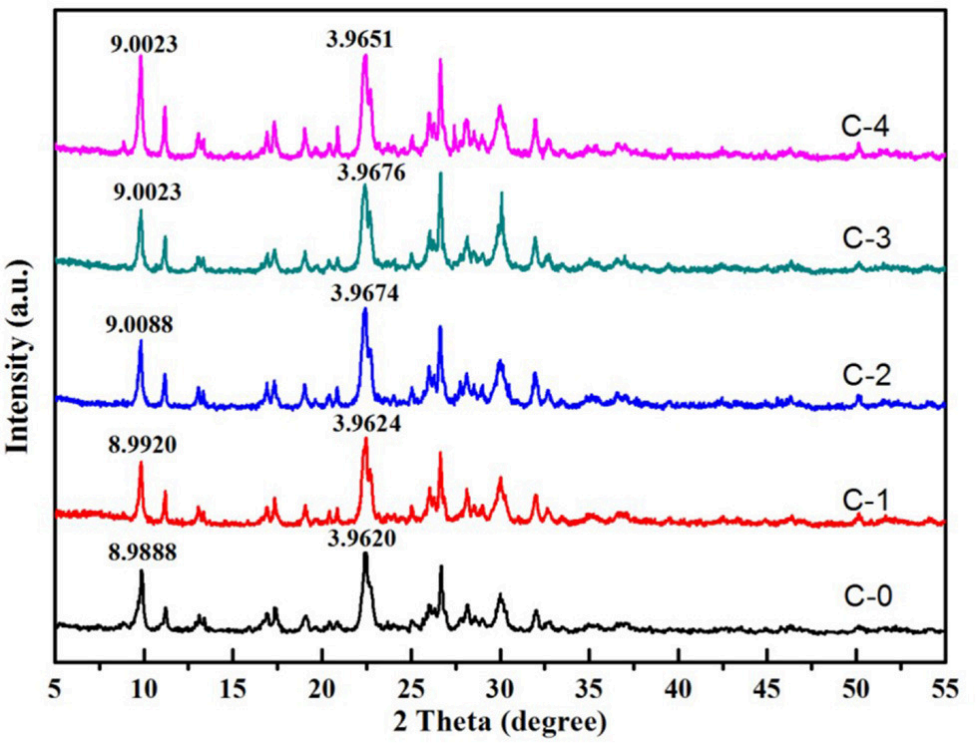
XRD of clinoptilolites.

The XRD patterns were mainly used for confirmation the difference in all clinoptilolites without and with treatment using NaCl solution (Figure 4). The major mineral in all samples was clinoptilolite with minor amounts of quartz. All the lattice parameters were not observably influenced by the different contents of NaCl. We couldn't see the characteristic peak of NaCl in modified clinoptilolites, It showed that the Na^+^ entered the lattice, and we combined the chemical composition analysis in Table 2. So we know it was replaced with Ca^2+^. The obvious difference was the D value of the characteristic peak was smaller than modified clinoptilolites. It was because the exchange of different ions leaded to the change of lattice spacing.

**Table 2:** BET of modified clinoptilolites treated by NaCl of different concentrations with N_2_ at 77 K.

	**C-1**	**C-2**	**C-3**	**C-4**
BET (m^2^/g)	16.1	16.0	16.0	15.9
External surface area (m^2^/g)	12.0	11.8	12.1	11.8
Micropore surface area (m^2^/g)	4.0	4.2	3.9	4.1
Vp (m^3^/g)	0.068	0.065	0.062	0.073
D (nm)	16.8	16.2	15.5	17.7

**Figure 5:**
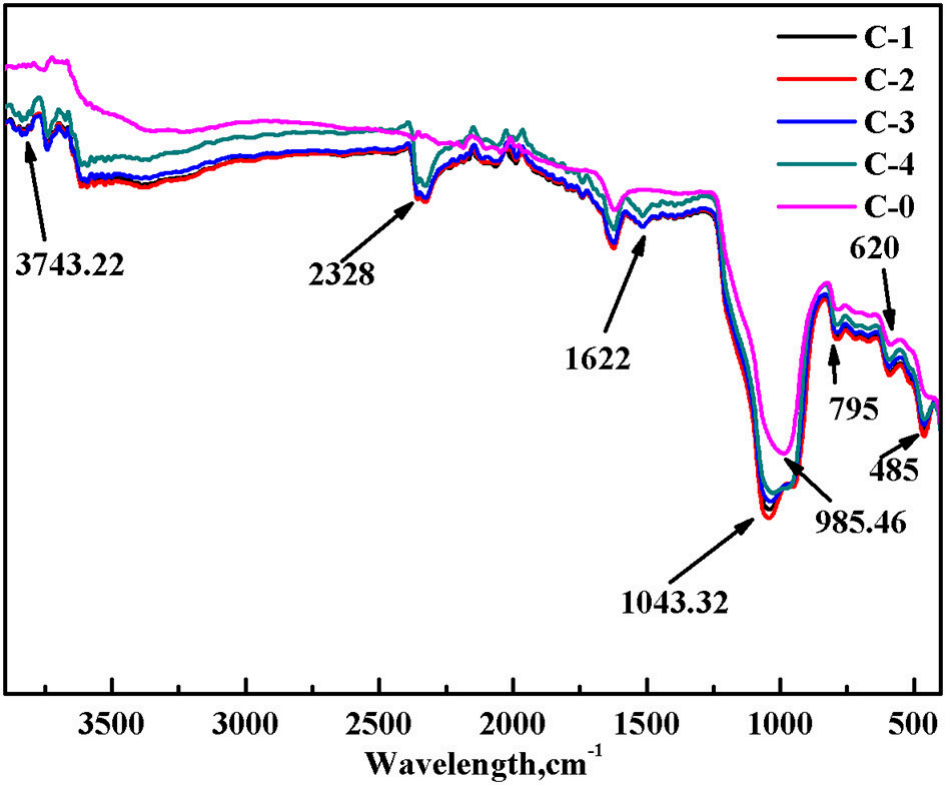
FT-IR spectra of modified clinoptilolites.

The broad band at 620 cm^−1^ in the clinoptilolites spectra were attributed to stretching vibrations related to Si-O tetrahedron structure (Figure 5). The characteristic peak of clinoptilolite without ion-exchange was weak. Furthermore, the vibration absorption peak of Si-O-Si and Al-O-Si appears at 795 cm^−1^. Compared with clinoptilolite without ion-exchange, some evident changes in characteristic absorption peaks were discovered. The characteristic peak at 985.46 cm^−1^ deriving from Al-O vibrations shifted to 1043.32 cm^−1^, caused a small amount of Al-O losing after ion-exchange. A small account of non-framework Al stuck in the unit cell was shifted by ion-exchange. And a small account of Al was shifted from framework because of Al-O-Si hydrolyzing in the clinoptilolites. Then cavities were formed leading to pore volume increasing. For modified clinoptilolites, the Si-O and/or Al-O out-of-plane bend occurred at 485 and 1,622 cm^−1^. Thus, the intensity of Al-O out-of-plane bend in modified clinoptilolites was stronger than that of clinoptilolite. It was because ion-exchange caused a small amount of Al-O losing. The broad band at 2,328 cm^−1^ mainly resulted from stretching vibrations of OH^−^ groups on modified clinoptilolites after ion-exchange. The broad band at 3,743 cm^−1^ mainly resulted from stretching vibrations of O-H on modified clinoptilolites after ion-exchange.

**Figure 6:**
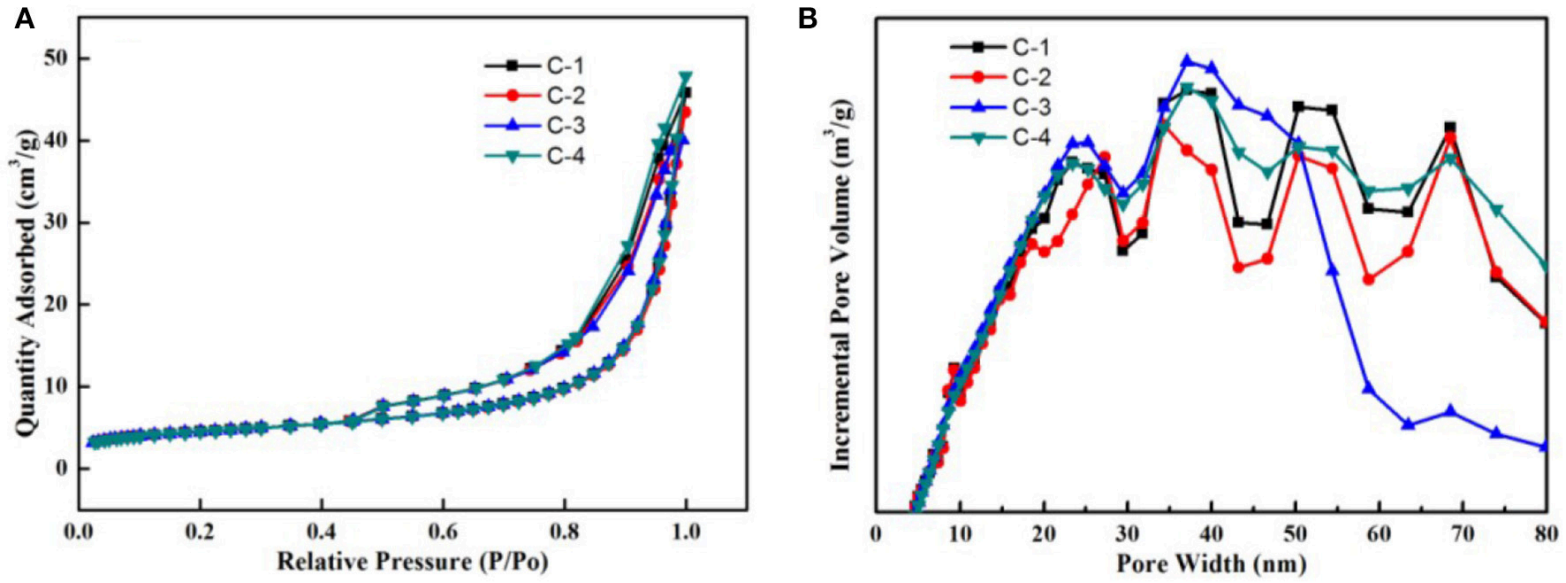
**(A)** N_2_ adsorption-desorption isotherms at 77 K; **(B)** pore size distributions.

The N_2_ adsorption-desorption isotherms at 77 K with the four adsorbents were shown in Figure 6A. According to the IUPAC classification, all curves were identified as type IV. The surface has mesopore and macropore, The curve of p/p0 region of low relative pressure is convex up, in the higher p/p_0_ region, the adsorbed material is condensed by capillary, the isotherm obtained by desorption does not coincide with the isotherm obtained by adsorption, and the desorption isotherm lags over the adsorption isotherm. So they present a hysteresis loop. It belongs to the class D loop. It is mainly due to the slit holes formed by sloping sheet stacking. In Figure 6B, we know most of the pores are between 5 and 80 nanometers, They have different pore structure by loading different contents of sodium ions. According to Table 2, we know that the internal surface area of C-2 adsorbents is the largest. With the increase of the loading of sodium ions, the micropore surface area of adsorbent increased first and then decreased. So the micropores are adjusted. At the beginning, the calcium ion was replaced by sodium ion, and the pore channel of clinoptilolite became smaller. When loading a certain amount, a large number of sodium ions loaded the surface of clinoptilolite and blocked some channels.

### Simulated Experiments

To explore the separation capability of CH_4_/N_2_ on the modified clinoptilolites, the simulated static experiments were conducted using BET equipment, and the simulated dynamic experiments were conducted using the device of adsorption of single tower (Figure 1), in which pure N_2_ and CH_4_ gas were chosen as gas-supply.

**Figure 7:**
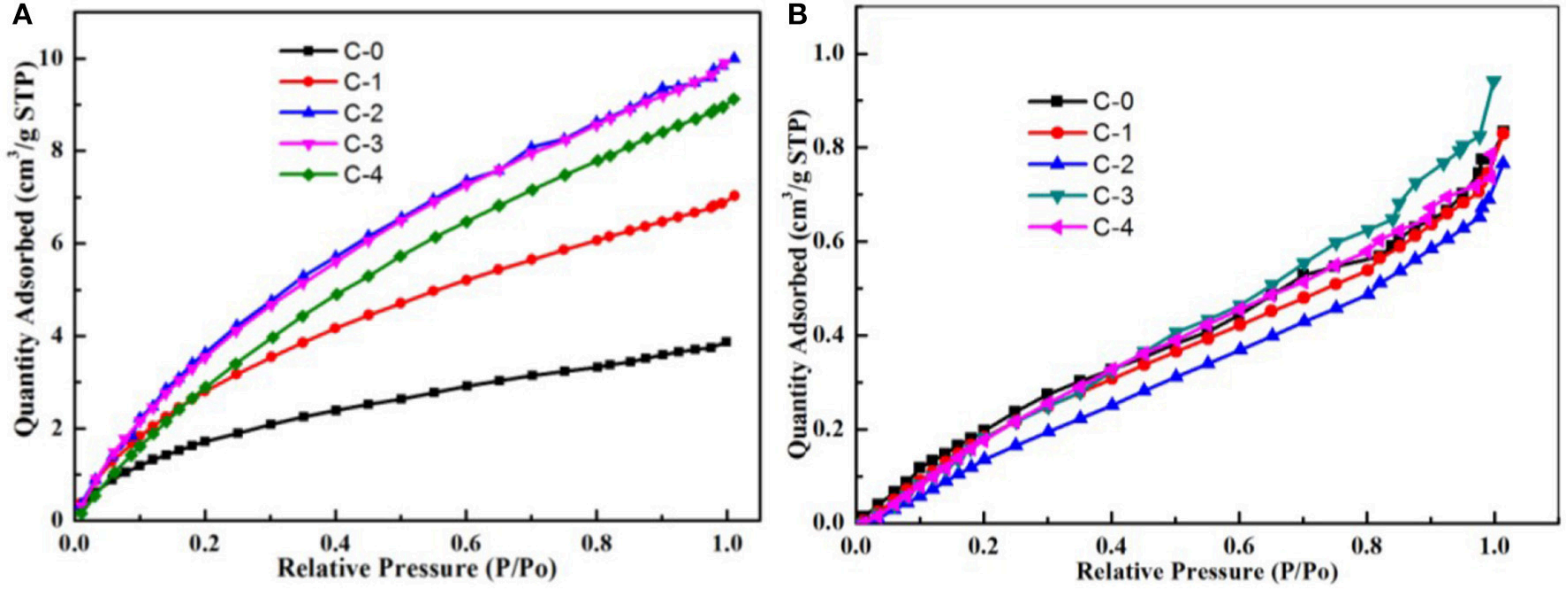
**(a)** N_2_ adsorption of the simulated static experiments at 298 K; **(b)** CH_4_ adsorption of the simulated static experiments at 298 K.

CH_4_ adsorption and N_2_ adsorption of the simulated static experiments were shown in Figure 7. It should be noted that adsorption of the two molecules is competitive and thus the gas-supply in the simulated static experiments is high purity N_2_ and high purity CH_4_ at 298 K, separately. Before the adsorption experiment begun, The absorbents were vacuum activated for 8 h at 370°. From Figure 7, there was obvious difference between quantity adsorbed of CH_4_ and N_2_ using clinoptilolites. At the same relative pressure condition, quantity adsorbed of N_2_ on clinoptilolites was much more than that of CH_4_. From Figure 7A, the quantity adsorbed of N_2_ using clinoptilolites followed: C-3 = C-2> C-4> C-1> C-0. From Figure 7B, the quantity adsorbed of CH_4_ using clinoptilolites was as follows: C-3> C-4> C-0> C-1> C-2. Considering the contradiction between adsorption capability of clinoptilolites for CH_4_ and N_2_, it is obvious that the C-2 adsorbents static equilibrium separation coefficient is the larger than other three. It is the most potential adsorbent for separating CH_4_/N_2_.

**Figure 8:**
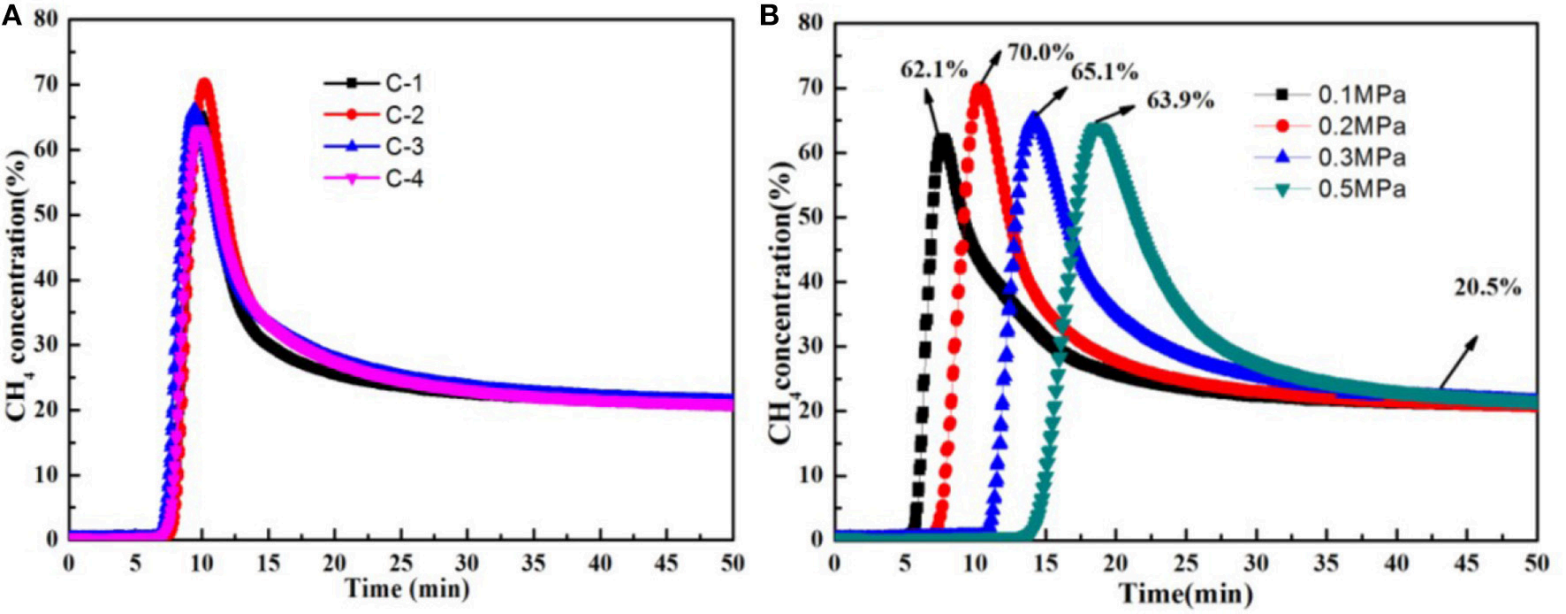
**(A)** The different adsorbents breakthrough curve of nitrogen adsorption at 298 K on 0.2 Mpa, **(B)** The C-2 breakthrough curve of nitrogen adsorption at 298 K on 0.1, 0.2, 0.3, 0.5 Mpa.

Dynamic experiments had been done. The CH4 volume concentration of product is obtained at 298 K on certain pressure when the feed gas is a mixture of CH_4_ (20%) and N_2_ gas (80%), as shown in Figure 8. Concentrated CH_4_ could be obtained directly by using these adsorbents of C-1, C-2, C-3, C-4. In this experiment, before testing, the adsorbent was vacuum activated for 8 h at 648 K, and then the package was sealed for use. When the concentration of CH_4_ of the top of the tower is 20%, it is put back to normal pressure, and a 30 min vacuum is activated and regenerated. The experiment was repeated three times, and the data are recorded in the third experiment. Figure 8A showed the different adsorbents breakthrough curve of nitrogen adsorption at 298 K on 0.2 Mpa. In the dynamic adsorption curve, it showed that CH_4_ concentration can be increased.C-1 is from 20 to 65.2%, C-2 is from 20 to 70.0%, C-3 is from 20 to 66.1%, C-4 is from 20 to 63.1%. Moreover, they can be continuously regenerated. So the adsorbent of C-2 is the best among these adsorbents, which is consistent with the static adsorption results. Figure 8B shows the C-2 breakthrough curve of nitrogen adsorption at 298 K on the different pressure. We can control residence time of raw gas in adsorbent by adjusting the pressure of carrier He gas. The residence time corresponding to 0.1, 0.2, 0.3, and 0.5 Mpa is 5, 7.5, 11, and 14 min, respectively. The peak value of CH_4_ reaches 70.0% when the residence time is 7.5 min. The results show that the N_2_ adsorption was bigger than CH_4_ when the mixture of CH_4_/N_2_ were in the absorbing tower. The bigger the pressure, the longer the residence time. CH4 adsorption was bigger than N2 adsorption when they were adsorbed. The separation factors would decrease. So, It is very important to choose the suitable residence time.

From the above, it seemed that the C-2 shows the greatest performance at 648 K under 0.2 MPa within 50 min, in comparison to the other modified clinoptilolites, as a underlying sorbent in kinetic PSA processes for the N_2_/CH_4_ separation. The different concentrations of Na^+^ that were existed in its porous network as well as their distribution were the primary influence factor that specifies the adsorption and kinetic properties of the clinoptilolites. Thus, the ion-exchange with differences in the concentration of Na^+^ disturbed the Na^+^ distribution as well as the electrostatic field inside the clinoptilolite's pores affecting the adsorption property.

## Conclusion

The material structure and CH_4_/N_2_ adsorbability of raw and Na^+^ ion-exchanged clinoptilolites have been examined in detail using PSA. The effect of adsorbent prepared by clinoptilolite with different sodium ion content on methane nitrogen separation is very different. The clinoptilolite adsorbents can be adjusted for their pore channel by controlling the loaded amount of sodium ions.

The C-2 adsorbent prepared using 0.2 mol/L NaCl solutions was the most promising for the kinetic PSA separation of CH_4_/N_2_, giving the better adsorptivity and influence factors concerning the CH_4_ separation.

From the simulated static experiments, it indicated that N_2_ and CH_4_ are both competent in diffusing into the clinoptilolites while N_2_ adsorptions of clinoptilolites are more excellent. The pertinent results also indicated that adsorption capability of ion-exchanged clinoptilolite using 0.2 mol/L NaCl solutions was the optimal adsorbent for separating CH_4_/N_2_ at low pressure, considering the contradiction between adsorption capability of clinoptilolites for CH_4_ and N_2_.

According to the simulated dynamic experiments, the ion-exchanged clinoptilolite using 0.2 mol/L NaCl solutions exhibits the best performance at 648 K under 0.2 MPa within 50 min, in comparison to raw and other modified clinoptilolites, as a underlying sorbent in kinetic PSA processes for the N_2_/CH_4_ separation.

The ion-exchange with differences in the concentration of Na^+^ as well as the electrostatic field inside the clinoptilolite's pores affecting the adsorption property. Finally, further manipulation for CH_4_ separation of the clinoptilolite is underway with coal bed gas as gas-supply.

## Author Contributions

XH and ZL conceived and designed the project. XH performed the experiments and wrote the manuscript. HH, XL, and YH analyzed the data.

### Conflict of Interest Statement

The authors declare that the research was conducted in the absence of any commercial or financial relationships that could be construed as a potential conflict of interest.
